# Hertfordshire sarcopenia study: design and methods

**DOI:** 10.1186/1471-2318-10-43

**Published:** 2010-06-29

**Authors:** Harnish P Patel, Holly E Syddall, Helen J Martin, Claire E Stewart, Cyrus Cooper, Avan Aihie Sayer

**Affiliations:** 1 Academic Geriatric Medicine, University of Southampton, Southampton General Hospital, Southampton, UK; 2 Medical Research Council Epidemiology Resource Centre (MRC ERC), University of Southampton, Southampton General Hospital, Southampton, UK; 3 Institute for Biomedical Research into Human Movement and Health, Manchester Metropolitan University, UK; 4 Institute of Musculoskeletal Sciences, University of Oxford, UK

## Abstract

**Background:**

Sarcopenia is defined as the loss of muscle mass and strength with age. Although a number of adult influences are recognised, there remains considerable unexplained variation in muscle mass and strength between older individuals. This has focused attention on influences operating earlier in life. Our objective for this study was to identify life course influences on muscle mass and strength in an established birth cohort and develop methodology for collection of muscle tissue suitable to investigate underlying cellular and molecular mechanisms.

**Methods:**

One hundred and five men from the Hertfordshire Cohort Study (HCS), born between 1931 and 1939 who have historical records of birth weight and weight at one year took part in the Hertfordshire Sarcopenia Study (HSS). Each participant consented for detailed characterisation of muscle mass, muscle function and aerobic capacity. In addition, a muscle biopsy of the vastus lateralis using a Weil-Blakesley conchotome was performed. Data on muscle mass, function and aerobic capacity was collected on all 105 participants. Muscle biopsy was successfully carried out in 102 participants with high rates of acceptability. No adverse incidents occurred during the study.

**Discussion:**

The novel approach of combining epidemiological and basic science characterisation of muscle in a well established birth cohort will allow the investigation of cellular and molecular mechanisms underlying life course influences on sarcopenia.

## Background

Skeletal muscle is central to physical as well as metabolic capability however, sarcopenia, the loss of muscle mass and strength with age impairs the maintenance of these capabilities into later life. Sarcopenia is associated with multiple adverse outcomes for the older person from impaired mobility and disability [1,2] morbidity from impaired glucose tolerance as well as diabetes [3], falls [4], fractures [5], and contributes to increased health care costs [6] and mortality [7,8]. The predicted rise in the number of older people over the age of 65 in the next two decades highlights the need for the increased understanding of important influences and consequences as well as research into developing preventive and treatment strategies for sarcopenia [9].

Declines in muscle strength are driven, in part, by a reduction in muscle mass which in turn is mediated by a global reduction in myofibre number and myofibre atrophy [10,11]. There are several well known non-modifiable and modifiable factors that influence adult skeletal muscle mass and strength. These include age, gender, adult body size, heritability, physical activity and nutrition [12-16]. However, there remains considerable unexplained variation in both muscle mass and strength in older people that leads to the question of whether influences occurring earlier in the life course such as developmental influences are important in determining adult muscle mass and strength. This life course approach potentially widens the window for beneficial interventions in contrast to the current focus, which is on later life [17].

Observational epidemiological evidence exists for developmental influences on muscle mass and strength in middle aged as well as older adults, and has shown that small size at birth (a proxy marker for an adverse intrauterine environment) is associated with reduced muscle mass and strength [18-21]. Associations have been demonstrated between size at birth and reduced fat free mass using anthropometric indices [20], size at birth and reduced muscle mass determined by urinary creatinine excretion [22], body composition DXA [23], as well as directly measured size using pQCT [24]. The associations between size at birth and muscle strength have been seen in a few studies. For example, those conducted in the Hertfordshire Cohort [19], in a middle-aged group of men and women born in 1946 participating in the National Survey of Health and Development [25] and in a Finnish cohort of men and women born between1934 and 1944 [21]. Pooled data derived from 10 studies that have investigated the relationship between lower birth weight and reduced grip strength have revealed remarkable homogeneity in this association with an effect size estimate of a 2.06 kg increase in grip for every kg increase in birth weight [26].

Interest is now focusing on the mechanisms underlying these epidemiological associations and current research aims to investigate whether the relationships are mediated through skeletal muscle fibres, capillary density and/or through alterations in anabolic or catabolic intracellular signalling pathways. Muscle fibre number is a critical determinant of muscle mass and strength [27]. There is strong evidence from animal studies for the effect of an adverse intrauterine environment on the subsequent number of myofibres in the fetus [27]. The majority of these studies have use models of prenatal nutritional manipulation with the observed effects on subsequent myofibres dependent on the timing, nature and severity of the insult.

Results from an ovine study showed peri-implantational and late gestational maternal under nutrition differentially affected myofibre density and function in fetal triceps muscle [28]. Both nutritional insults resulted in decreased myofibre and capillary density with late gestational under nutrition predominantly causing a reduction in type I, slow twitch, myofibre density. The effect of under-nutrition on myofibre development and birth weight has been seen in other animal models and there is evidence that the effect of fewer myofibres has detrimental effects on muscle mass and meat quality in post-natal life [29-32].

Molecular signals that govern hypertrophy include the intracellular signalling molecules phosphatidylinositol-3-phophate kinase, Akt and mammalian target of rapamycin (PI3K/Akt/mTOR). These are part of a key signalling pathway activated by upstream ligands, such as IGF-1, that regulate myoblast proliferation in utero [33] as well as muscle protein synthesis and hypertrophy in post-natal life through increased protein transcription [34]. Conversely, inflammatory mediators such as IL-6 and TNF-α can directly cause myocyte loss through activation of the apoptotic pathways or through activation of the E3 ubiquitin ligases Murine Ring Finger-1 (MuRF-1) and Muscle Associated F-box (MAFBx/Atrogin-1) that are involved in protein degradation [35].

There is limited evidence for an association between small size at birth and change in muscle morphology or imbalance in the anabolic and catabolic intracellular signalling pathways in human adults. One study of 20 low birth weight and 20 normal birth weight men aged 19 years demonstrated a change in muscle fibre composition and size [36]. Fewer type IIa (fast twitch) fibres were seen (absolute fibre-count mean 43.4 vs 63.2 fibres, p = 0.004) as well as an increase in type IIa fibre mean area (7444 μm^2 ^vs 5754 μm^2^, p = 0.014) in the lower birth weight group compared to higher birth weight group [46]. No statistically significant differences were seen in type IIx fibre number, area or, type I fibre number although the type I fibres tended to be larger in the LBW group (5660 μm^2 ^vs 4848 μm^2^). The authors suggest altered muscle morphology may contribute to insulin resistance and type II diabetes in the low birth weight men later in life [36]. Another study investigating the relationship between size at birth and muscle morphology in a group of 27 adult women showed no changes in myofibre density, capillary density or muscle enzymatic activity [37]. There is observational evidence for the effect of inflammation, mediated through cytokines such as IL-6 and TNF-α, as a contributing factor to sarcopenia [38-40] and that inflammation in adult life may be potentiated by poor growth in early life [41].

The evidence from animal studies as well as the findings from the study in young men with low birth weight needs to be taken forward in studies of older people to investigate the cellular and molecular mechanisms underlying the developmental origins of sarcopenia. This study takes the novel approach of collecting muscle tissue in addition to data on muscle mass and strength in an established birth cohort. A description of the study design and methods now follows.

## Design/methods

### Study design

**Figure 1:**
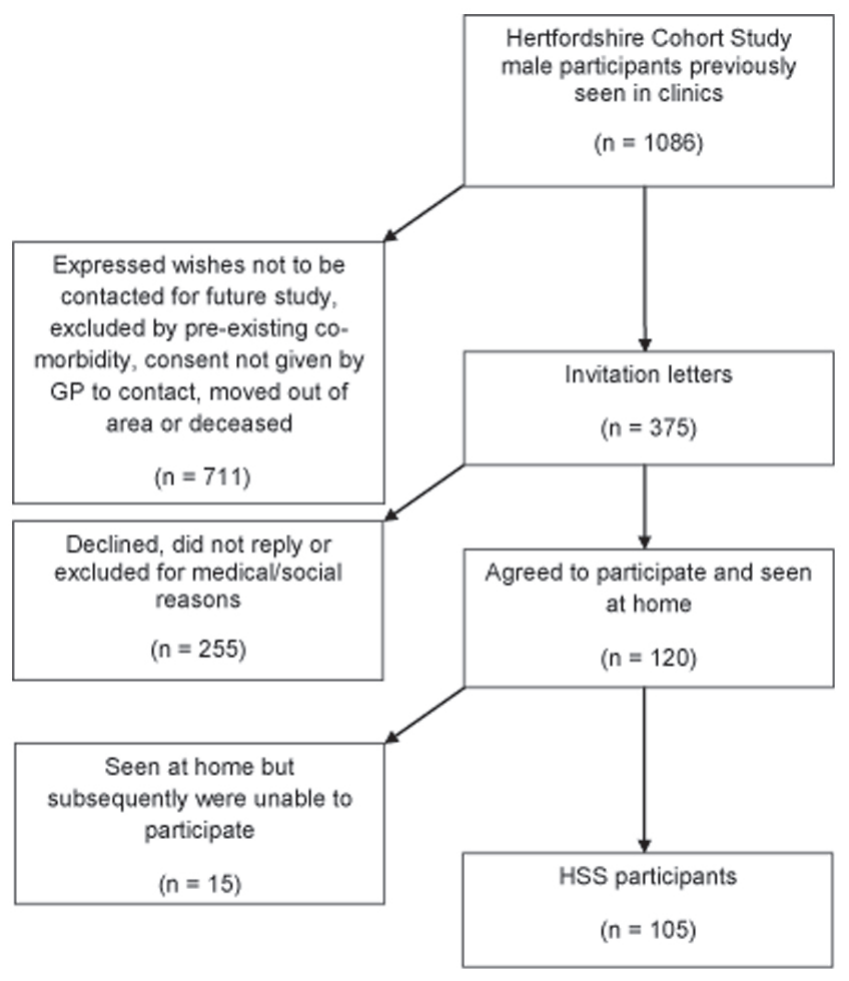
**Flow chart for the recruitment into the Hertfordshire Sarcopenia Study**.

The Hertfordshire Sarcopenia Study (HSS) is a retrospective cohort study designed to investigate life course influences on muscle morphology, mass and strength in community dwelling older people. It is part of the established Hertfordshire Cohort Study (HCS) that comprises a total of 2997 men and women born in Hertfordshire between 1931 and 1939 and has previously been described in detail [42]. From this total, 1086 men were identified as potential HSS participants. After several tiers of exclusion as detailed in Figure 1, a total of 375 men were invited to take part. Each participant's GP was approached to ascertain the appropriateness of taking part in HSS. One hundred and twenty participants were visited at home by the study physician where requirements for the study were explained in detail, written informed consent obtained and a health and activity questionnaire administered. An appointment was then made to attend the Wellcome Trust Clinical Research Facility in Southampton General Hospital for further investigations that involved an overnight stay. One hundred and five men took part in the study and were fasted overnight prior to their arrival to the research facility (Figure 1). The study received ethical approval from the Hertfordshire Research Ethics Committee (number 07/Q0204/68).

### Inclusion/exclusion criteria

Inclusion criteria for the study were availability of historical records of weight at birth and at one year. Participants were excluded if they had a prior diagnosis of diabetes mellitus, ischaemic heart disease, myopathy or any neuromuscular disorder that affected the legs. In addition, participants who were on anticoagulants such as warfarin were excluded. Female participants were not included in this phase of the study.

## Clinical measurements

### Muscle biopsy

**Figure 2:**
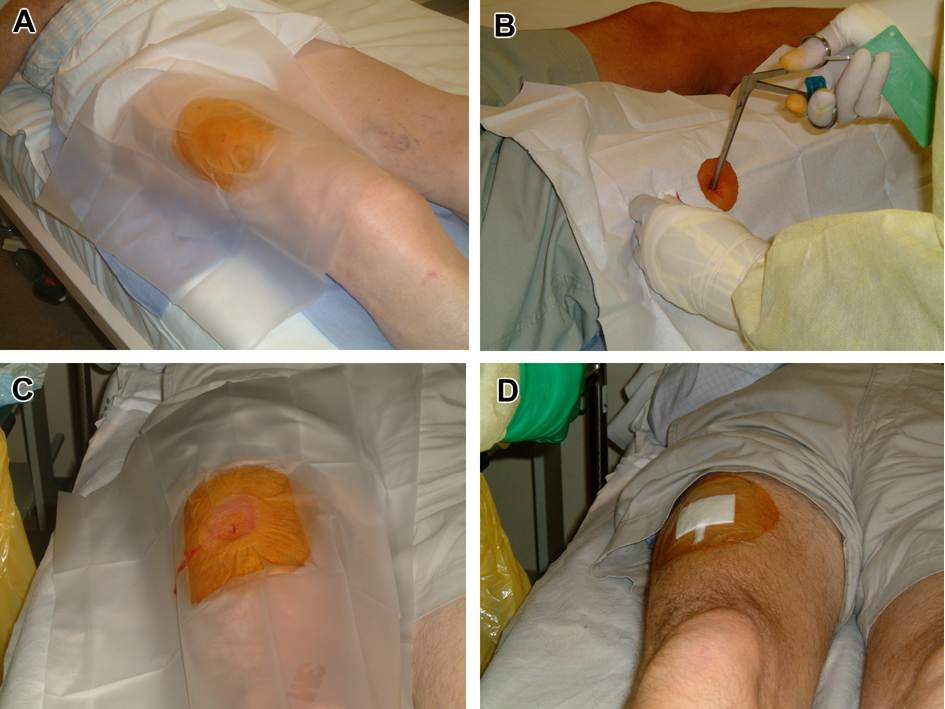
**The muscle biopsy procedure**. A. The leg was exposed from the groin crease down to the ankle. The biopsy area over the vastus lateralis was shaved of hair, marked and isolated with a sterile drape that had a 10 cm aperture B. After the skin and subcutaneous tissue was infiltrated with local anaesthetic, the skin was punctured with a size 11 scalpel down to the fascia and the conchotome was inserted into the track made by the scalpel. The conchotome was rotated through 90° to cut the muscle C. Sustained pressure for 5 minutes was applied over the 5-10 mm incision to minimise bleeding D. The wound was closed with steri-strips and was dressed with absorbent dressing.

The participants were fasted overnight prior to muscle biopsy. The semi-open muscle biopsy was performed using a Weil Blakesley conchotome with a 6 mm biting tip (Gebrüder Zepf Medizintechnik, Dürbheim, Germany) (Figure 2) [43]. The left or right vastus lateralis muscle was biopsied over the antero-lateral aspect of the thigh. This site, approximately two-thirds down a line from the anterior superior iliac spine to the patella, was readily accessible and did not contain an overlying neurovascular bundle. Following biopsy retrieval, the wound was closed with steri-strips and an absorbent dry dressing was placed over the wound after which, a two-layer compression bandage was applied for 6 hours. The procedure took approximately 20 minutes to complete. Participants were allowed to mobilize 30 minutes after the procedure (Figure 2).

### Tissue preparation and fixation

The biopsy tissue was divided and either processed for morphological studies, or stored at -80°C for future molecular study (e.g. gene expression studies). With respect to morphological study, one sample piece was orientated longitudinally under a dissecting microscope and placed in 5 ml of cold acetone and allowed to fix overnight in a freezer at -20°C before being embedded in Glycol Methacrylate (GMA) [44] for later immunohistochemical analyses (myofibre type, number, density [fibres/mm^2^], area [μm^2^] and capillary density [capillaries/mm^2^]). One sample was also orientated longitudinally and fixed overnight at 4°C in 3% glutaraldehyde and 4% formaldehyde solution before processing in resin for subsequent electron microscopy studies.

### Dual energy X-ray absorptiometry (DXA)

A body composition DXA scan (Hologic Discovery, auto whole body software version 12.5) was performed on all participants to quantify regional as well as total lean mass, fat mass and bone mineral content.

### Anthropometry

Weight was measured once to the nearest 0.1 kg. Height was measured on a stadiometer (SECA, Hamburg, Germany). A flexible, circumference measuring tape was used to measure mid-upper-arm circumference, waist circumference; at a point mid-way between the costal margin and the iliac crest in the mid-axillary line, hip circumference; at the widest part of the hip between the greater trochanter and the lower buttock level. Mid-thigh circumference was measured at a point half way between the protuberance of the greater trochanter and a circumferential line drawn at the upper border of the patella. All measurements were read to the nearest 0.1 cm. Skinfold measurements were taken in triplicate from the triceps, biceps, sub-scapular and upper supra-illiac regions using a standard calliper (Crymych, Holtain Ltd, UK). Measurements were all of the non-dominant side. Blood pressure was measured on the right arm with a digital device (DASH 3000, GE Medical systems, USA) and a standard 12 lead electrocardiogram was taken and interpreted to identify any occult ischaemic heart disease or cardiac arrhythmia.

### Grip strength

Isometric grip strength was measured three times in each hand, alternating between right and left hands, using a Jamar handheld hydraulic dynamometer (Promedics, UK) with the participant seated. The dynamometer was supported, but not held by the investigator during testing. The maximum of six measurements to the nearest 1 kg was used in subsequent analyses [45].

### Physical performance measures and aerobic fitness

A validated battery of tests was used to assess motor function that included: five timed chair rises, a 6 metre timed up-and-go where participants were asked to stand from sitting, walk 3 metres, turn around and return back to the seated position; customary walking speed over 3 metres, and standing balance, where participants were timed standing on one leg with eyes open [46-48]. A sub-maximal exercise test was performed on a stationary cycle ergometer. The starting workload was 25 watt, which was maintained for 3 minutes, thereafter the workload increased by 10 watts every minute. The test was stopped if the participants reached 80% of the maximum age predicted heart rate, a respiratory exchange ratio of 1.1; signifying their anaerobic threshold, or the participants wanted to stop. Gas parameters were measured for 3 minutes after stopping the test. Oxygen consumption (VO_2_), expired carbon dioxide (VCO_2_) and volume of expired air (VE) were determined through breath-by-breath analyses using a Metalyser 3B gas analyser system (Cortex Biophysik, Cranleigh and Co., Birmingham, UK) [49]. Heart rate was monitored through a Polar receiver (Polar Electro UK, Ltd).

### Blood collection

Fasting blood samples were taken from the anterior cubital fossa for subsequent glucose, insulin, HbA1c, hormonal, inflammatory and DNA analyses. A total of 40 ml were collected in two plain (10 ml each) lithium heparin (10 ml), EDTA (5 ml) and fluoride oxalate (5 ml) tubes (Vaccutainer Systems, Beckton Dickinson, UK). All except the EDTA tube were centrifuged at 2500 rpm for 15 minutes at 4°C. Respective serum, plasma and fluoride oxalate aliquots were prepared in 1 ml tubes and frozen at -80°C until further analyses. The supernatant from the EDTA tube was discarded and the residue (clot/whole blood) was pipetted into 1 ml aliquots and stored at -80°C until further analyses.

## Questionnaires

### Health and activity

A general health questionnaire was used to record details of smoking and alcohol habits, co-morbidity, medication use and lifestyle. The present questionnaire was based on questionnaires used previously in the cohort that had also ascertained participants' social class and dietary habits.

### Cognitive function

An AH4 test to determine intelligence quotient as well as the Mill Hill vocabulary test were administered to test decline in cognitive function [50].

### Study feedback

All participants were asked to provide feedback one week after the clinic visit. The feedback questionnaire consists of 12 anonymous questions about their experience of the study and specifically, their experience of the muscle biopsy.

## Investigations completed thus far

One hundred and twenty subjects consented to take part in the study (Figure 1). Home visits were arranged for all 120 participants where written informed consent was obtained, a health and activity questionnaire administered and WTCRF clinics arranged in Southampton. Fifteen participants withdrew after the home visit because of ongoing hospital investigations, time constraints or were caring for their spouse. Subsequently, 105 participants, with a mean age of 72.5 years were seen in WTCRF clinics over the course of 14 months (October 2007 - December 2008). Three participants were seen on any one clinic. The strength and body composition measures of the study sample are presented in Table 1. The mean height was 174.2 cm (range 157.6-193.7), mean weight 82.8 kg (58.1-118.8), body mass index (BMI) 27.2 kg/m^2 ^(18.6-38.3), birth weight 3.5 kg (2.3 - 5.4), weight at one year 10.3 kg (7.7-12.7), grip strength 38.7 kg (18-66). The total lean body mass was derived from DXA and was 56.5 kg (42.7-75.9); the appendicular skeletal mass represents the total lean mass of four limbs and was 24.2 kg (16.9-33.7). Fat mass was also calculated from DXA and was a mean of 22.7 kg (range 5.6-46.2).

**Table 1:** Hertfordshire Sarcopenia Study participant characteristics

	Study sample			
	n = 105			
	
	Mean	SD	Min	Max
Age (years)	72.5	2.5	68.3	77.4
Height (cm)	174.2	6.7	157.6	193.7
Weight (kg)	82.8	12.6	58.1	118.8
BMI (kg/m^2^)	27.2	3.6	18.6	38.3
Birth weight (kg)	3.5	0.7	2.3	5.4
Weight at one (kg)	10.3	1.1	7.7	12.7
Grip strength (kg)	38.7	8.4	18	66
Total body lean mass (kg)_DXA_	56.5	6.7	42.7	75.9
Appendicular skeletal mass (kg)_DXA_	24.2	3.2	16.9	33.7
Fat mass (kg)_DXA_	22.7	6.8	5.6	46.2

One hundred and two muscle biopsies were successfully obtained with a Weil Blakesley conchotome using a standard aseptic protocol. The three participants who were not eligible for the biopsies were on treatment that could have influenced wound healing (n = 2) or predispose to haematoma formation (n = 1). The mean biopsy mass from 53 samples was 107 mg (range 20 to 290 mg) - sufficient for subsequent histological, electron microscopy and molecular studies.

Anthropometry, measures of muscle function (grip strength and physical performance) and venesection was completed on all 105 participants according to protocol. Eighty-four participants completed the sub-maximal exercise test: ten participants did not complete the test fully and 11 participants were excluded from the test (ECG abnormality n = 6 or were on medication that artificially lowered heart rate e.g. β blockers, n = 5).

All 105 participants completed the health and activity questionnaires and 98 participants completed both the AH4 and Mill Hill tests. Ninety-three participants provided complete feedback questionnaires. Thirty-four (37%) of the participants found the biopsy procedure better than expected, 52 (56%) as they had expected and 7 (7%) worse than expected. All participants were mobile within half an hour of the procedure without any difficulty and 60 (65%) of the participants resumed their normal daily activity the next day (but avoided vigorous activity). Thirty-three (35%) participants were back to their usual activity after two days. There were no serious wound complications and there was full healing within a week. Eighty-five (91%) of the participants were willing to have the biopsy again for research purposes [43].

No adverse incidents occurred during the course of the study. All participants received detailed feedback on clinically relevant parameters (ECG, bone mineral density and blood pressure) that were also fed back to their General Practitioners.

## Statistical analyses

All data will be analysed using STATA release 10 (STATA Corp, Texas, 2009). Normality of variables will be assessed using visual inspection of histograms and tests of skewness and kurtosis. Variables will be log_e _transformed as necessary. Variables will be summarised using means and standard deviations (SD), medians and inter-quartile ranges (IQR) for continuously distributed variables and frequency and percentage distributions for categorical variables. The relationships between birth weight and muscle morphology mass and function will be principally explored using the Student's t-test or analysis of variance (ANOVA), and linear regression. Multiple linear regression will be used to adjust for the potential confounding influence of age, anthropometry, physical activity, lifestyle, co-morbidity and medication use.

The relationships between life course influences and muscle morphology, mass and function will be explored using the Student's t-test, ANOVA or Pearson's correlation coefficient followed by linear and multiple regression. The inter-relationships between muscle morphology, mass and strength will similarly be determined using Pearson's correlation coefficient and linear regression.

### Sample size

Sample size calculations assumed a standard deviation of 22.0 for type IIa fibre number based on a previous study in young men and showed that a study of 50 lower birth weight and 50 higher birth weight men would have 80% power, at the 5% significance level, to declare means of type IIa fibre number of 56 and 45 between groups statistically different.

## Discussion

There is a growing body of epidemiological evidence supporting an association between birth weight and adult mass and strength. However the underlying mechanisms are not known and there are few studies in this area. One study involved a small number of older women and another, 40 young men. In contrast the HSS involves over 100 older men and future research will include both Hertfordshire men and women. Previous experience from the Hertfordshire Physical Activity Trial suggests that healthy community dwelling older women are willing to have a muscle biopsy alongside more routine assessment of body composition [51]. No adverse complications arose as a result of the muscle biopsy in this study.

This novel study will allow the determination of potential cellular and molecular mechanisms that underlie the evidence linking life course influences and sarcopenia. This will be achieved through study of muscle tissue obtained by biopsy in addition to obtaining usual measurements on muscle mass, strength and aerobic fitness in a group of community dwelling older men aged 68 to 76 years with records of birth weight and weight at one year. We have already shown that muscle biopsy of the vastus lateralis by the conchotome technique is both feasible and acceptable in this group [43]. The study findings will not only consolidate our understanding of the changes in muscle that occur with age and their relationship to early growth but will also have the potential to inform the development of beneficial interventions to implement across the life course.

## Abbreviations

HCS: Hertfordshire Cohort Study; HSS: Hertfordshire Sarcopenia Study; DXA: Dual-energy x-ray absorptiometry; pQCT: peripheral quantitative computerised tomography; MuRF-1: Murine Ring Finger -1; MAFbx: Muscle Associated F-box; IGF-1: Insulin like Growth Factor -1; WTCRF: Wellcome Trust Clinical Research Facility; GMA: Glycol Methacrylate; EDTA: ethylenediaminetetraacetic acid; HbA1c: Glycosylated haemoglobin; VO_2_: oxygen consumption; VCO_2_: volume of expired carbon dioxide; VE: volume of expired air.

## Competing interests

The authors declare that they have no competing interests.

## Authors' contributions

HPP: Study physician and principal investigator, HES: Medical statistician, HJM: Physical performance measures, CES: Molecular analyses, CC, AAS: HCS principal investigators. All authors read and approved the final manuscript.

## Pre-publication history

The pre-publication history for this paper can be accessed here:

http://www.biomedcentral.com/1471-2318/10/43/prepub
